# Nanomechanical Signatures in Glioma Cells Depend on CD44 Distribution in IDH1 Wild-Type but Not in IDH1R132H Mutant Early-Passage Cultures

**DOI:** 10.3390/ijms24044056

**Published:** 2023-02-17

**Authors:** Mikhail E. Shmelev, Vladislav M. Farniev, Nikita A. Shved, Vadim V. Kumeiko

**Affiliations:** 1 Institute of Life Sciences and Biomedicine, Far Eastern Federal University, 690922 Vladivostok, Russia; 2 A.V. Zhirmunsky National Scientific Center of Marine Biology, Far Eastern Branch, Russian Academy of Sciences, 690041 Vladivostok, Russia

**Keywords:** glioma, AFM, nanomechanics, biomechanics, cell stiffness, CD44, malignancy, cell proliferation, primary cell cultures, IDH1

## Abstract

Atomic force microscopy (AFM) recently burst into biomedicine, providing morphological and functional characteristics of cancer cells and their microenvironment responsible for tumor invasion and progression, although the novelty of this assay needs to coordinate the malignant profiles of patients’ specimens to diagnostically valuable criteria. Applying high-resolution semi-contact AFM mapping on an extended number of cells, we analyzed the nanomechanical properties of glioma early-passage cell cultures with a different IDH1 R132H mutation status. Each cell culture was additionally clustered on CD44+/− cells to find possible nanomechanical signatures that differentiate cell phenotypes varying in proliferative activity and the characteristic surface marker. IDH1 R132H mutant cells compared to IDH1 wild-type ones (IDH1wt) characterized by two-fold increased stiffness and 1.5-fold elasticity modulus. CD44+/IDH1wt cells were two-fold more rigid and much stiffer than CD44-/IDH1wt ones. In contrast to IDH1 wild-type cells, CD44+/IDH1 R132H and CD44-/IDH1 R132H did not exhibit nanomechanical signatures providing statistically valuable differentiation of these subpopulations. The median stiffness depends on glioma cell types and decreases according to the following manner: IDH1 R132H mt (4.7 mN/m), CD44+/IDH1wt (3.7 mN/m), CD44-/IDH1wt (2.5 mN/m). This indicates that the quantitative nanomechanical mapping would be a promising assay for the quick cell population analysis suitable for detailed diagnostics and personalized treatment of glioma forms.

## 1. Introduction

Glioma is a malignant brain tumor that is clinically separated by its morphology, mutation status, and aggressivity. The big problem of its diagnostics and treatment is intracerebral localization, which usually prohibits the pre-operational biopsy obtaining that is typically substituted with indirect diagnostic methods [1]. Usually, the first cellular and molecular investigations may be provided only after hours or days after surgery. It might be useful to develop ex-vivo diagnostics using biopsy materials captured after tumor resection. These samples can be utilized for further instant analyses on live brain slices or dissociated cells using traditional histological or immunochemical techniques supplemented with some innovative approaches. Atomic force microscopy (AFM) has recently burst into biomedical research, providing quantitative morphological and nanomechanical analysis via high-throughput physical mapping of tissues and cells.

Malignant cells are often softer than healthy cells. Generally in carcinogenesis, more aggressive cells tend to be less rigid than less aggressive cells [2,3,4]. Otherwise, cancer invasion propagated by extended cell migratory ability displays relatively higher cell rigidity deciphered in terms of Young’s modulus and stiffness [5].

The main components that determine the nanomechanical properties of tumors are the proteins of the entire tissue skeleton, including nuclear scaffold and cytoskeleton, cell junctions, and extracellular matrix (ECM)**.** F-actin, alpha- and beta-tubulin, and intermediate filaments modulate surface fluidity and shear response together with a core of cell nanomechanical pattern shaped by nuclear envelope, scaffold, and chromatin [6,7,8,9,10]. Cell nanomechanical properties and nanoarchitectonics reflect cell division and migration. Membrane hardness is specified by membrane cytoskeleton formation, organization, and assembly. Svitkina et al. (2018) described various forms of submembrane actin network, which are modified in a response on mechanical stress on cell surface [11]. Cellular matrix reshaping and tension combine to activate the Hippo pathway and YAP/TAZ DNA binding [12]. The nanomechanical signatures could be specific for highly proliferative or migratory cell phenotypes that are of interest as a tumor specification method [13].

The establishment of primary cell cultures from a tumor material could be a valuable approach for personalized therapy. Cultivated tumor cells are usually very heterogeneous by the phenotype and the degree of differentiation; this could be investigated by detecting the cell membrane proteins including the clusters of differentiation (CDs). In pancreatic carcinomas, the presence of the CD44 protein is usually associated with a cell stemness and increased malignancy [14]. According to the Cancer Genome Atlas (TCGA), expression of CD44 is most often associated with a low survival and bad prognosis. This effect was shown for many different tumors [15,16]. It is also known that the expression of this protein is combined with an increase in the expression of beta-actin [17]. It was not verified for the case above, but typically, high actin expression leads to an increase in cells’ Young’s modulus and stiffness. Recent papers have exhibited some interactions between cell mechanics and proliferation in association with migratory ability. Prior to move in blood vessels cells are characterized by lower amounts of adhesion molecules and, therefore, a lesser ability to adhere [18]. Activated metastatic tumor cells, after they enter the bloodstream, induce their softness and adhesion. This mechanism allows them to effectively spread through the body, surpass the biological barriers, and stick to healthy tissue [19].

Precise morphological and nanomechanical analyses applying AFM could be performed on live cells and tumor slices. Recently, we have elaborated a new AFM-based technique suitable for the both quantitative and qualitative assays on live tissue slices, which could be performed even intraoperatively to clarify the diagnosis [20,21]. However, tumor mechanical properties may be altered due to calcification and/or cicatricial rearrangement in ECM [22]. These changes in tissue mechanical properties may corrupt the AFM analysis of the proper cells, which is required to establish primary cell cultures to analyze cancer cell mechanics in terms of personalized treatment strategy.

Early studies specified that nanomechanical properties of gliomas depend on the IDH1 R132H mutation. Primarily, a non-direct method for tissue mechanics in vivo investigation utilizing magnetic resonance elastography displayed the IDH1 R132H gliomas as more rigid than the wild-type ones [23]. Direct nanomechanical in vitro examination uses the lentiviral overexpression of IDH1 wild-type and IDH1 R132H mutant alleles in U-251MG, LN-229, and U-343MG standard cell lines [24]. Standard cell lines artificially overexpressing normal or mutant IDH1 are interesting experimental models, but in terms of clinical diagnostics, each case requires the proof of primary cell cultures with naturally occurring allele variants diversified on different cell phenotypes affecting each other [25]. Gliomas have highly heterogeneous cell populations, involving only 2–3 percent of cells with the highest division ability, which are called glioma stem cells (GSCs) [26]. The standard cell lines do not have such heterogeneity and may form several specific characteristics, distinguishing them from intact tumor cells [27].

In this study, we compared the early-passage cell cultures derived from patients’ glioma samples, which were different in their IDH1 R132H mutation status supplemented with dissimilar CD44 expression, to find the peculiar nanomechanical signatures characterizing different brain cancer cells.

## 2. Results

### 2.1. Cell Culture Establishing and Description

The established cell cultures were taken from patients’ samples gathered in the course of tumor resection and designated as BT1, BT32, BT39, and BT40. The tumor samples were characterized in the hospital with the conventional histological technique supplemented with IHC. We performed genetic analysis to identify the most frequent allele variants associated with different gliomas in accordance with the 2021 WHO recommendations [28,29]. According to these results, all samples were characterized as adult-type diffuse gliomas; BT32 and BT39 samples bear an IDH1 R132H mutant allele and correspond to grade III astrocytoma, respectively. BT1 and BT40 were grade IV glioblastomas with an IDH1 wild-type (Table 1).

**Figure 1:**
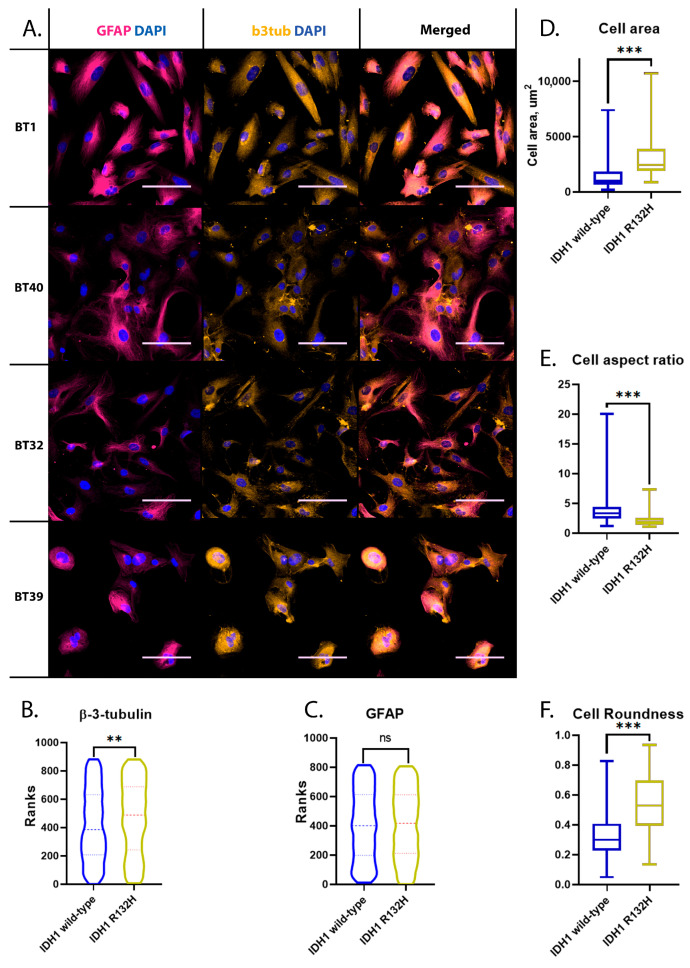
Immunocytochemical and morphological characterization of obtained glioma early-passage cell cultures. Asterisks indicate level of statistical significance: ** *p* ≤ 0.01, *** *p* ≤ 0.001 and “ns” *p* > 0.05. (**A**) GFAP and beta-3-tubulin immunostaining. Scale bar is 100 um. (**B**) Beta-3-tubulin labeling intensity comparison in IDH1 wild-type and IDH1 R132H mutant early-passage cell cultures. (**C**) GFAP labeling intensity comparison in IDH1 wild-type and IDH1 R132H mutant early-passage cell cultures (**D**) Surface area comparison of IDH1 wild-type and IDH1 R132H cell cultures. (**E**) IDH1 wild-type and IDH1 R132H cell aspect ratio comparison. (**F**) Cell roundness of IDH1 wild-type and IDH1 R132H cultures.

To verify the cell phenotypes in the established primary cell cultures, an additional immunocytochemical analysis was conducted. All of the cell cultures were characterized by a glial phenotype displaying specific a GFAP distribution that was manifested significantly in IDH1 wild-type cell cultures (Figure 1 BT1, BT40) compared to IDH1 mutant variants (Figure 1 BT32, BT39). Additionally, beta-3-tubulin expression was found in all cell cultures, which is characteristic for glial cancer cells in contrast to normal ones (Figure 1) [30]. To quantify the differences in beta-3-tubulin and GFAP expression in IDH1 wild-type cell cultures and IDH1 R132H cell cultures, a two-tailed Mann-Whitney U test was conducted. IDH1 R132H cell cultures were characterized by significantly higher beta-3-tubulin expression than IDH1 wild-type ones, with an approximate *p*-value of 0.0025 (Figure 1B), and there were no significant differences in GFAP expression between these cell culture groups (*p* value 0.77) (Figure 1C).

**Figure 2:**
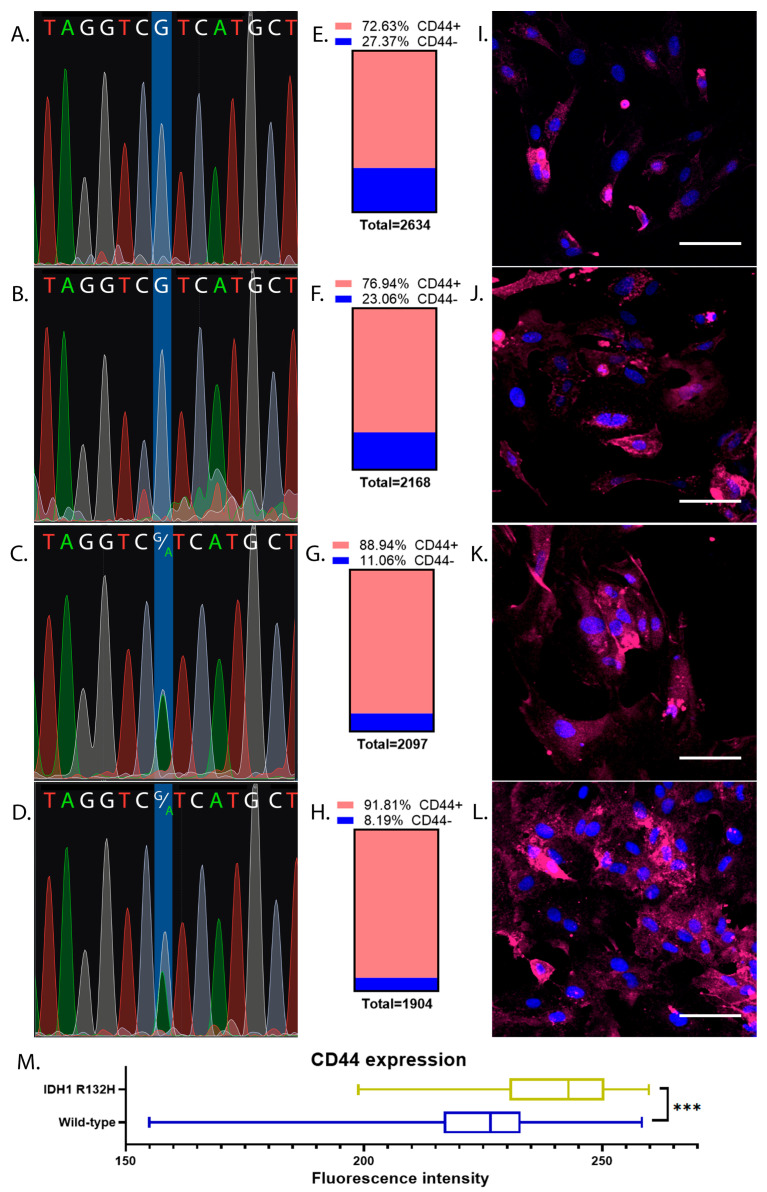
IDH1 sequence chromatograms and CD44 immunostaining of early-passage glioma cell cultures (P-4). Laser scanning microscopy with equal image acquisition parameters. Blue color is for DAPI, and red is for CD44. Scalebar is 100 um. (**A**) Sequence chromatogram for BT1 cell culture, IDH1 wt; (**B**) Sequence chromatogram for BT40 cell culture, IDH1 wt; (**C**) Sequence chromatogram for BT32 cell culture, IDH1 R132H mutant; (**D**) Sequence chromatogram for BT39 cell culture, IDH1 R132H mutant; (**E**) Fraction of CD44+ cells in BT1 cell culture; (**F**) Fraction of CD44+ cells in BT40 cell culture; (**G**) Fraction of CD44+ cells in BT32 cell culture; (**H**) Fraction of CD44+ cells in B391 cell culture; (**I**) CD44 labeling of BT1 cells; (**J**) CD44 labeling of BT40 cells; (**K**) CD44 labeling of BT32 cells; (**L**) CD44 labeling of BT39 cells; (**M**) CD44 labeling intensity comparison of IDH1 wild-type and IDH1 R132H mutant early-passage cell cultures. Asterisks indicate level of statistical significance: *** *p* ≤ 0.001.

The mutant cells had a more fibroblast-like morphology, while the IDH1 wild-type had a pronounced elongated fusiform shape (Figure 1A,D–F). IDH1 R132H cells had a more than two-fold larger cell area, with a median value of 2441 um^2^ (IQR, 1981 um^2^) in comparison to the IDH1 wild-type median area of 980.9 um^2^ (IQR, 1175.7 um^2^). The differences between the groups were confirmed by a two-tailed Mann-Whitney U test with a *p*-value less than 0.0001 (Figure 2D). Differences in cell shape were approved by cell aspect ratio and roundness calculations (Figure 2E,F). IDH1 mutant-type cells were defined with a spread morphology with a low aspect ratio of 1.9 (IQR, 1.1) and a higher roundness of 0.53 (IQR, 0.3). In contrast to the IDH1 mutant-type, the IDH1 wild-type demonstrated a pronounced elongated fusiform shape with a cell aspect ratio of 3.3 (IQR, 1.9) and a roundness of 0.3 (IQR, 0.18). The two-tailed Mann-Whitney U test proved the difference between cell culture groups in terms of the cell aspect ratio and roundness with a *p*-value below 0.0001 (Figure 1E,F).

After primary cell culture propagation, additional sequencing was applied for early-passage secondary cultures (*p*-4) to prove that the cell cultures did not change their characteristic genotypes throughout the course of the spontaneous cell selection engaged by genome instability during subcultivation (Figure 2A–D). BT1 and BT40 remained IDH1 wild-type genotypes (Figure 2A,B). BT32 and BT39 were also characterized by the IDH1 R132H genotype corresponding to the initially determined tumor genotype (Figure 2C,D).

CD44 immunostaining showed the predominance of CD44+ cells among the populations of all four glioma cultures (Figure 2E–L). All established early-passage glioma cell cultures were characterized by the presence of a large amount of CD44+ cells; 7194 cells from the 8803 totally investigated were CD44+. In BT1, a cell culture of 1913 cells from a total of 2634 were CD44+ (Figure 2E); in BT40 cell culture, 1668 cells from 2168 were CD44+ (Figure 2F); in BT32 cell culture, 1865 cells from 2097 were CD44+ (Figure 2G); in the BT40 cell culture, 1748 cells from 1904 were CD44+ (Figure 2H). The total percentage of CD44+ cells in IDH1 R132H cell cultures corresponded to 88.94% and 91.85% for BT32 and BT39, respectively, which was relatively higher than the wild-type ones, which displayed 72.63% and 76.94% for BT1 and BT40, respectively. To prove the differences in CD44 labeling between IDH1 R132H mutant- and IDH1 wild-type cells, a two-sided Fisher’s exact test was conducted. The obtained *p*-value was significant at the level of *p* < 0.0001. To quantify the differences between IDH1 wild-type cell cultures and IDH1 R132H cell cultures in CD44 expression, a two-tailed Mann-Whitney U test was conducted. Mutant cells were characterized by significantly higher CD44 immunostaining (*p*-value < 0.0001) (Figure 2M).

### 2.2. IDH1 R132H Is Characterized by Induced Rigidity and Stiffness but a Lower Proliferation Rate

**Figure 3:**
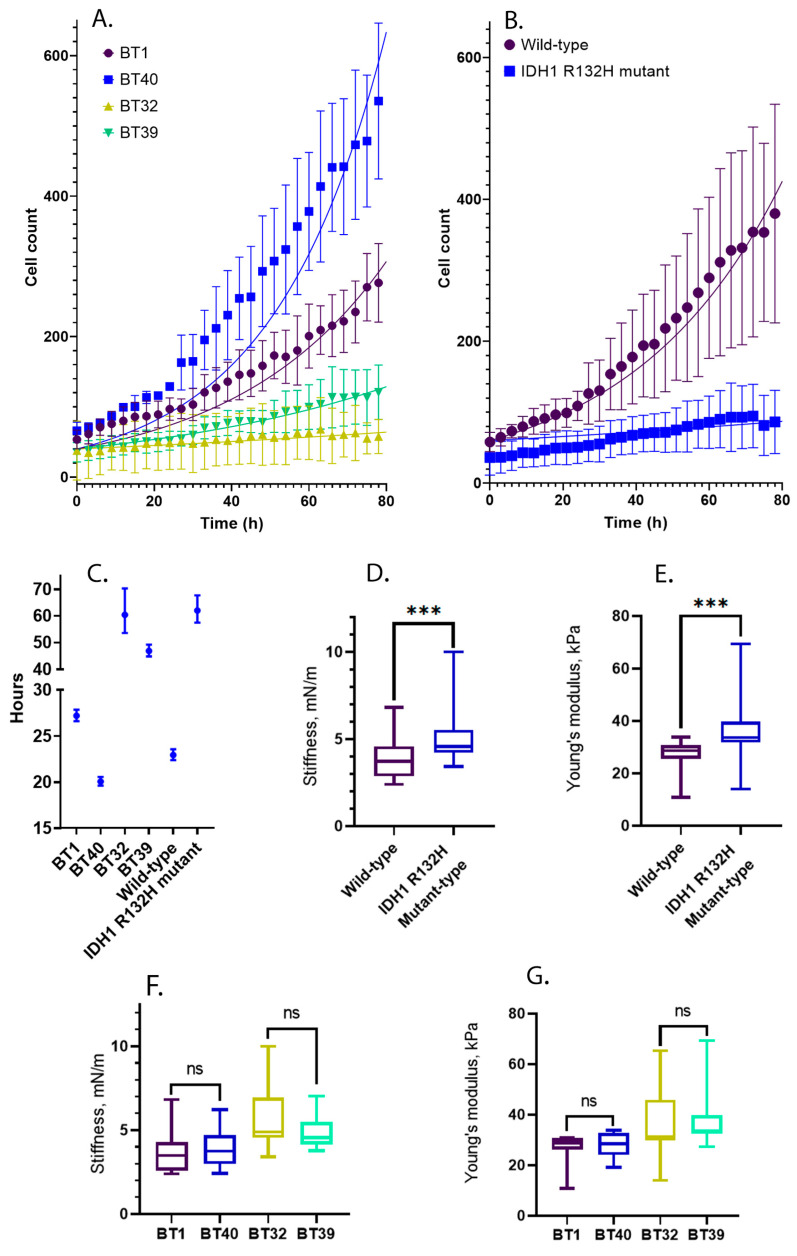
Cell proliferation rate for R132H mutant and wild type cell cultures. Asterisks indicate level of statistical significance: *** *p* ≤ 0.001 and “ns” *p* > 0.05. (**A**) Growth curves for the each analyzed cell culture; (**B**) Growth curves for the cell cultures grouped by the presence of IDH1 R132H mutations; (**C**) Cell doubling time calculated for the each analyzed cell culture and clustered cell cultures by the presence of IDH1 R132H mutation; (**D**) The comparison of the stiffness collected from whole populations of the cells from the IDH1 R132H cell cultures vs. wild type cell cultures. (**E**) The comparison of the Young’s modulus collected from whole populations of the cells from the IDH1 R132H cell cultures vs. wild type cell cultures. (**F**) The comparison of the cell stiffness measured from specific cell lines. (**G**) The comparison of the cell Young’s modulus measured from specific cell lines.

The established cell cultures are characterized by different proliferation rates. To quantify the differences between the cell cultures and find the cell doubling times, the raw data was fitted by using non-linear regression exponential (Malthusian) growth (Figure 3A,B). The wild-type cell cultures were characterized by the highest proliferation rate and the lowest doubling time of 20.07 h for BT40 and 27.21 h for BT1 cell cultures. The IDH1 R132H mutant cell cultures showed a low proliferation rate and a large cell doubling time of 46.85 h for BT39 and 60.40 h for BT32 cell cultures (Figure 3A,C). The cell doubling times calculated for all IDH1 R132H mutant cell cultures were 62.02 h and 22.95 h for wild-type ones (Figure 3B,C). Additionally, to quantify the differences in cell growth, a two-way ANOVA analysis was conducted. All cell cultures displayed significantly different proliferation rates (*p* < 0.0001).

We found that IDH1 R132H mutant cells in early-passage secondary cultures are more rigid than IDH1 wild-type ones. The stiffness and the Young’s modulus of both IDH1 R132H mutant cell cultures median values were 4.7 mN/m (IQR, 1.3) and 33.718 kPa (IQR, 10.881), respectively, which was larger than those parameters in both IDH1 wild-type cell cultures, where the stiffness median value was 3.7 mN/m (IQR, 1.8), and the Young’s modulus median value was 28.59 (IQR, 8.287). To prove the statistical significance of the differences between cell culture groups, the two-tailed Mann-Whitney U test was conducted separately to compare the Young’s modulus and stiffness values of IDH1 R132H and IDH1 wild-type cell cultures. The obtained *p*-values were less than 0.0001 for both pairs; hence, there were significant differences between the groups. (Figure 3D,E).

To quantify the differences between cell cultures with a similar genotype, a non-parametric ANOVA test was performed. According to the results of Dunn’s multiple comparisons test, there were no significant differences between the two investigated IDH1 R132H cell cultures; the Young’s modulus median values were 31.27 kPa (IQR, 15.974) and 33.71 kPa (IQR, 7.367), the *p*-value was 0.73, the stiffness median values were 4.9 mN/m (IQR, 2.7) and 4.5 mN/m (IQR, 1.2) for BT32 and BT39, respectively, and the *p*-value was 0.25 (Figure 3F,G). The nanomechanical signatures in both IDH1 wild-type cell cultures were also fairly similar without significant differences. The Young’s modulus median values were 26.31 kPa (IQR, 9.842) and 28.67 kPa (IQR, 7.344), the *p*-value was bigger than 0.99; the stiffness median values were 3.2 mN/m (IQR, 1.7) and 3.7 mN/m (IQR, 1.5) for BT1 and BT40, respectively (*p* > 0.99) (Figure 3F,G).

### 2.3. CD44+ Cells Are More Rigid Than CD44- Cells in IDH1 Wild-Type but Not in IDH1 R132H Mutant Gliomas

To provide an appropriate dataset, high-resolution AFM mapping was performed to reach more than 100 cells for each genotype. CD44+ cells accounted for 69% in the IDH1 wild-type population and 92% among the IDH1 R132H mutant cells that were taken in the AFM investigation.

**Figure 4:**
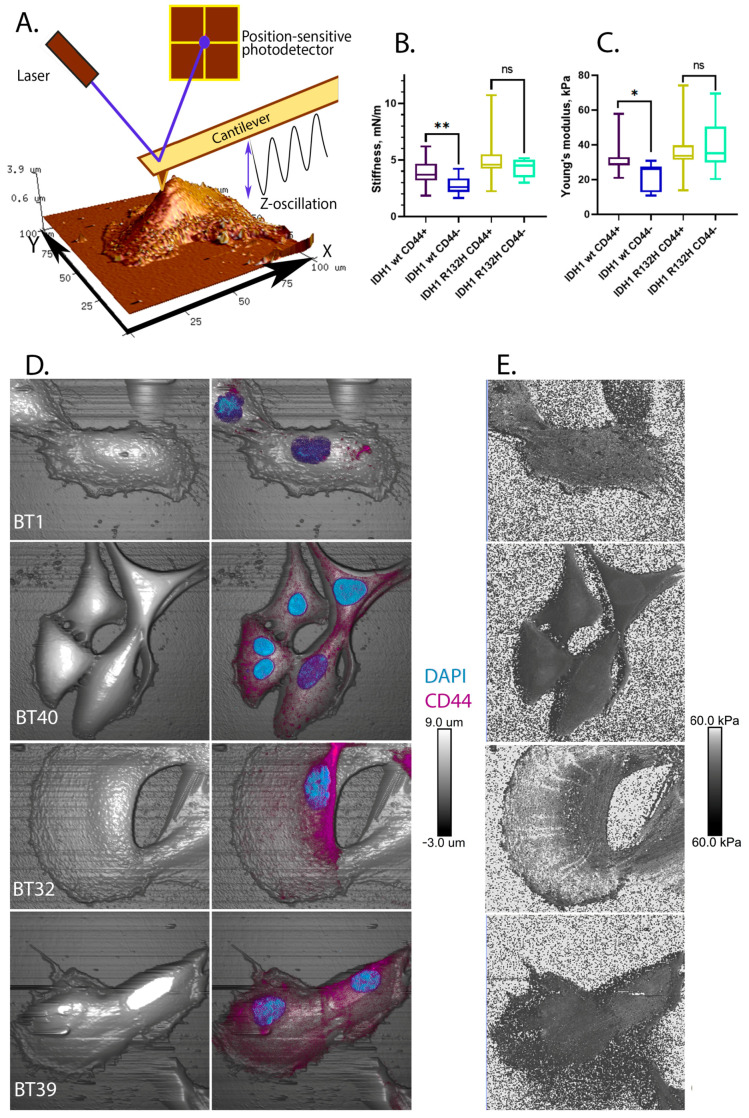
Nanomechanical referent images of each cell culture. Asterisks indicate level of statistical significance: * *p* ≤ 0.05, ** *p* ≤ 0.01 and “ns” *p* > 0.05. (**A**) Atomic force microscopy working principle. (**B**) The distribution of stiffness in cell culture groups depending on their genotype and CD44+/−; (**C**) The distribution of Young’s modulus in cell culture groups depending on their genotype and CD44+/− (**D**) The composite nanomechanical and ICC characterization of cell cultures; (**E**) Young’s modulus mapping of referent cells in each cell culture.

We found that the presence of CD44 on IDH1 wild-type cells is associated with increased mechanical rigidity, which reaches values close to those of IDH1 R132H mutant cells by using atomic force microscopy assay on living adherent cells (Figure 4A,D). The calculated Young’s modulus median value of the IDH1 wild-type CD44+ cells was 28.67 kPa (IQR, 8.406 kPa), and the stiffness median value was 3.7 mN/m (IQR, 1.43 mN/m), which is higher than that of the IDH1 wild-type CD44 cell population, in which the Young’s modulus was 25.98 (IQR, 14.731 kPa), and the stiffness was 2.54 mN/m (IQR 1.16 mN/m) (Figure 4B,C). Dunn’s multiple comparisons test also showed a significant difference between the mean ranks, which were 48.35 for the Young’s modulus values (*p*-value 0.01) and 59.92 for the stiffness values (*p*-value 0.005). The IDH1 wild-type cultures (BT1 and BT40) have been characterized as soft ones with poorly developed cortical cytoskeletons, while the IDH1 R132H mutant cultures (BT32 and BT39) are more rigid, and their cytoskeleton structures are well-identified on the cell surface and possess high stiffness (Figure 4B,E).

We found that all mutant cell lines have significantly homogeneous stiffness and Young’s modulus parameters in the groups determined by cell labeling, and the mutant cells are significantly stiffer than the wild-type ones (Figure 4B,C).

According to the Dunn’s multiple comparisons test results, the IDH1 R132H mutant cells are significantly homogeneous without any dependance on CD44 membrane expression (mean rank difference is 10.80 for stiffness (*p*-value 0.97) and 3.070 for Young’s modulus (*p*-value > 0.99)). This may explain the contradiction in that mutant cells are characterized by a higher level of CD44 expression but, at the same time, a reduced invasiveness and malignancy of these tumors. An increased level of CD44 expression has a greater effect on the malignancy of wild-type cells compared to IDH1 mutant cells.

## 3. Discussion

In this study, glioma primary cell cultures derived from patients were obtained, and their successful subcultivation allowed us to propagate early-passage secondary cultures that were classified according to their phenotype and genotype. We found that there were two cultures without marker mutations recommended by the WHO for glioma differential diagnostics. Two other cell cultures bear an IDH1 R132H mutation that is usually associated with a better clinical outcome.

Applying immunocytochemical analysis, we proved the glial phenotype of established cell cultures by a high intensity of GFAP and beta-3 tubulin immunostaining. Interestingly, the differential labeling of IDH1 R132H mutant- and wild-type cells for CD44 surface marker was found. According to the Human Protein Atlas, CD44 protein is widespread in the human brain, including the glial cells [31]. This protein plays an important role in brain development and functioning, including cell differentiation, migration, spreading, and inflammatory regulation [32,33,34,35]. Glioma tissue is characterized by significantly high immunoreactivity with anti-CD44 antibody compared to normal brain tissues and an increased amount of hyaluronic acid, up to twenty times more than in healthy adult brain; that is akin to the fetal central nervous system [31,36].

In low-grade gliomas, elevated CD44 is associated with poor prognosis [37]. Patients with low expression of CD44 in glioma have better clinical outcomes [38]. In high-grade gliomas, including glioblastomas, CD44 also may serve as an effective marker for detailed diagnosis and clinical prediction. The one-year survival rate is significantly lower for patients with glioblastoma multiforme displaying high CD44 immunostaining [39]. 

CD44 is a protein whose membrane expression is associated with the most aggressive cells in tumor and may be recognized as an additional prognostic marker for various malignant tumors [40,41]. Cancer cell invasiveness in carcinomas and gliomas is typically mediated by epithelial-mesenchymal transition, which is responsible for the generation of relatively low-proliferating phenotypes with a high ability to migrate [41]. High CD44 expression significantly correlates with the expression of drug resistance and metastasis-associated genes and generally occurs during an epithelial-mesenchymal transition [42]. According to the immunohistochemical analysis on tumor sections, CD44+ cells range from 60% to 90% of the population in glioma [43,44]. 

Both established early-passage IDH1 R132H cell cultures displayed a larger amount of CD44+ cells, reaching more than 90% in contrast to the IDH1 wild-type cell cultures characterized by 77% CD44+ cells as a maximum value. Glioma stem cells exhibit an increased level of CD44 [45]. In addition to that, our recent data, obtained by single nucleus transcriptomics of glioma stem cells captured from tumor samples by sorting for Sox2 positive nuclei, demonstrated that IDH1 R132H migrating astrocyte-precursor cells are characterized by the highest CD44 expression among different subpopulations of glioma stem cells [46].

IDH1 R132H mutant cell cultures are generally attributed to reduced proliferation rate in comparison to the IDH1 wild-type cell cultures. The U87MG cell line with induced IDH1 R132H mutation displayed higher time in the wound-healing assay than the IDH1 wild-type U87MG cells [47]. The presence of the IDH1 R132H mutation in glioma is associated with better clinical outcomes through enhancing sensitivity to temozolomide via regulation of the ATM/CHK2 pathway [48]. Our results for both established IDH1 wild-type cell cultures showed a relatively high proliferation rate with a doubling time of around 23 h, which is even faster than the U87MG cycling estimated as 30 h doubling time [49]. The mean value of the obtained early-passage IDH1 R132H glioma cell cultures’ doubling time was near 60 h, which is close to values obtained for the astrocyte cell line SVGp12 with lentiviral-induced IDH1 R132H mutation that required approximately 60 h to double the cell population [50].

An interesting finding was the nanomechanical distributions among cells in CD44+/− subpopulations. We found that the expression of CD44 in IDH1 wild-type cells is associated with a two-fold increase in hardness. However, subpopulations of CD44+ and CD44- cells in IDH1 R132H mutant cultures do not have a statistically significant difference in stiffness and Young’s modulus, but CD44+/IDH1 wild-type cells were still softer than IDH1 R132H mutant cells. The reason for cell hardening in wild-type cultures could be mediated by remodeling the cytoskeleton for directional migration leaded by hyaluronic acid [36,51,52,53]. The low hardness of the CD44+/IDH1R132H mutant cells may be explained by non-specific gene silencing caused by the synthesis of D-2-hydroxyglutarate in IDH1 R132H mutant cells, resulting in actin fibers’ aberrant assembling [54].

A possible explanation for the IDH1 R132H mutation effect on cell culture nanomechanics is that it may be an effect of AVIL protein, which is upregulated in high-grade glioblastoma, especially the IDH1 wild-type. This protein is crucial for GBM tumorigenesis via regulation actin folding and binding [55]. Another explanation for the IDH1 R132H mutant cell hardening may be Rac1-mediated cytoskeleton reorganization [56]. This protein is one of the hallmarks of tumor growth and metastasis and plays an important role in cell division and migration. Rac1 may induce actin synthesis and polymerization, which stimulate cell hardening and may enhance cell motility and mediate cell morphology transformations [57,58]. A soft cell surface with high membrane fluidity supports cell spreading and metastasis by facilitated migration through tissue membranes and barriers [24].

Cell invasiveness and metastatic potential could be detected by identifying their specific nanomechanical signatures [59,60]. We attempted to determine nanomechanical profiles that are characteristic for the investigated cell phenotypes. The increased stiffness and Young’s modulus of the IDH1 R132H mutant cells turned out to be highly indicative, which is probably mediated by both altered cytoskeleton and lipogenesis in mutant cells [24,46,61]. In this work, we first used the early-passage glioma cell cultures to compare hardness of IDH1 R132H mutant and IDH1 wild-type cells. We found that the mutant cell cultures displayed two-fold increase in stiffness and Young’s modulus. This fact can also explain the specific “spread-out” morphology of mutant cells in culture as compared to “directed” and “sprout” cells in IDH1 wild-type ones.

Thus, the distribution of cell nanomechanical properties may serve as the detailed diagnostic criteria for glioma clinical outcomes; assuming that CD44-/IDH1 wild-type cells display the lowest stiffness, CD44+/IDH1 wild-type are characterized by the moderate stiffness, and the IDH1 R132H mutant cell population is the stiffest one, mainly comprising CD44+ cells.

## 4. Materials and Methods

### 4.1. Primary Cell Culture Establishment

All tumor samples were provided by the Medical Center of the Far Eastern Federal University (FEFU) and depersonalized. The use of patient samples was approved by the FEFU Ethics Committee; all experiments were carried out in accordance with the principles of the WMA Declaration of Helsinki. All patients signed an informed consent document before participating in the study.

Cell culture establishment was performed by the following protocol:

A 3–5 mm tumor fragment was rinsed with sterile 1X PBS (Thermo Fisher, Waltham, MA, USA) solution to remove the blood traces.

The fragment was placed in a 100 mm cell culture dish (Eppendorf, Hamburg, Germany) with an addition of 3–5 mL of 1X trypsin-EDTA solution (Thermo Fisher, Waltham, MA, USA). After that, the tumor fragments were grinded by using sterile scalpel and tweezers until it became possible to aspirate them by using 1 mL pipette. The obtained suspension was transferred to a sterile 15 mL centrifuge tube (Eppendorf, Hamburg, Germany), closed, and placed for 20 min in a cell culture incubator with gentle stirring every 5 min.

To prepare the cell suspension for transferring to the cell culture flask, 10 mL of the media was added to the tube with the sample and carefully mixed by pipetting. Then the obtained suspension was transferred to a cell culture flask and put in a CO_2_ incubator for 24 h.

The first three passages after establishment cells were cultivated in serum-free Neurobasal medium (Thermo Fisher, Waltham, MA, USA) supplemented with EGF and FGF (Thermo Fisher, Waltham, MA, USA). Starting from the fourth passage, we used high-glucose Dulbecco’s Modified Eagle Medium (DMEM) (Thermo Fisher, Waltham, MA, USA) with 1 mM Sodium Pyruvate and 300 mg/L L-glutamine (Thermo Fisher, Waltham, MA, USA), supplied with 10% Fetal Bovine serum (FBS) (Thermo Fisher, Waltham, MA, USA), penicillin and streptomycin (Thermo Fisher, Waltham, MA, USA).

On the next day, the flask was rinsed with warm PBS, and fresh media were added.

Glioma cell culture was considered as established after two successful recultivations and GFAP labeling. Afterwards, a cell culture was labeled for the differentiation markers and snap-frozen in liquid nitrogen.

### 4.2. Cell Proliferation Assay

The established cell cultures were cultivated until the confluency grade became 80%; then, the cells were removed from the flask surfaces by using 0.25% trypsin solution (Thermo Fisher, Waltham, MA, USA) and reseeded on new flasks. As a cell culture media was used high-glucose DMEM with 1mM Sodium Pyruvate and 300 mg/L L-glutamine (Thermo Fisher, Waltham, MA, USA), supplied with 10% Fetal Bovine serum (FBS) (Thermo Fisher, Waltham, MA, USA), penicillin and streptomycin (Thermo Fisher, Waltham, MA, USA).

The migration and proliferation assays were considered by using a time-lapse Cell IQ machine, which is described by Narkilahti et al. [62]. This system acts as a hardware phase-contrast microscope and CO_2_ incubator assembly and a software complex designed for image recognition for the quantification of proliferation and migration image data.

### 4.3. Cell Culture Genotyping

The genomic DNA of cell cultures was isolated from cell culture samples containing 5 million cells per sample using the ExtractDNA Blood & Cells kit (Evrogen, Moscow, Russia) in accordance with the protocol provided by the manufacturer. 

Gene fragments containing regions of interest for IDH1 were amplified using DreamTaq Green PCR Master Mix (2X) (Thermo Fisher, Waltham, MA, USA). 

The PCR mixture was purified using the Cleanup Mini kit (Evrogen, Moscow, Russia). 

For Sanger sequencing, we used 10 ng of purified DNA by BigDyeTM Terminator v3.1 Cycle Sequencing Kit (Thermo Fisher, Waltham, MA, USA)

The sequencing reaction was purified using the BigDye XTerminatorTM Purification Kit (Thermo Fisher, Waltham, MA, USA) and sequenced on a 3500 genetic analyzer (Thermo Fisher, Waltham, MA, USA). 

The raw data results were analyzed using the SnapGene Viewer program (GSL Biotech, San Diego, CA, USA).

### 4.4. The Immunocytochemistry (ICC) and Immunohistochemistry (IHC)

Tumor sample immunohistochemistry analysis results were obtained from the clinical diagnosis.

The immunocytochemistry was performed by the following protocol.

Cells were fixed in 2% paraformaldehyde prepared on PBS for 15 min and washed 3 times for 5 min in 0.05% Tween-20 (Helicon, Moscow, Russia) prepared on PBS (PBS-T). The next step was membrane permeabilization by Triton X-100 (Helicon, Moscow, Russia) 0.5% solution prepared on PBS for 5 min at room temperature. After that, cells were incubated three times for 5 min in PBS-T to remove all traces of Triton X-100. To prevent nonspecific binding of antibodies, cells were incubated for 2 h with 3% Bovine Serum Albumin (Sigma-Aldrich, St. Louis, MO, USA) prepared on PBS. Before the addition of antibody solution, the cells were incubated three times for 5 min in PBS-T. 

Cell labeling was performed with primary beta-3-tubulin (T8578, Sigma-Aldrich, St. Louis, MO, USA), GFAP (G3893, Sigma-Aldrich, St. Louis, MO, USA), and CD44 (ab157107, Abcam, Cambridge, UK) in supply-recommended titer in PBS for 2 h at room temperature. To reduce antibody non-specificity, the cells were washed by incubation in PBS-T 3 times for 5 min before the labeling with secondary antibodies. We used Alexa Fluor 488 goat anti-rabbit IgG (H+L) (a11034, Thermo Fisher, Waltham, MA, USA) and Alexa Fluor 546 goat anti-mouse IgG (H+L) (a11003, Thermo Fisher, Waltham, MA, USA) secondary antibodies. The labeling procedure was performed for 1 h at room temperature. To reduce the non-specific fluorescence the 3-time incubation in PBS-T for 5 min was performed. To stain the nucleus, we added DAPI (Sigma-Aldrich, St. Louis, MO, USA) for 10 min with concentration of 300 nM in the final washing step.

CD44+ cells were calculated by the Imaris software.

Cell labeling for CD44 was performed after the AFM investigation to correlate the life cell AFM and CD44 labeling.

### 4.5. Protein Expression Quantification

ImageJ 1.53t software package was used to quantify the fluorescent images. Fluorescent images were acquired on similar image acquisition parameters. To subtract the image background, the threshold was chosen as a mean value of non-fluorescent part of image performed on each color channel. The mean fluorescence intensity was quantified after choosing a field as region of interest [63,64]. At least 200 cells of each cell culture were used to calculate the median fluorescence intensity. To compare the values between cell groups, non-parametric ANOVA tests (Kruskal-Wallis test and Dunn’s multiple comparison test) were performed.

### 4.6. Cell Morphology Analysis

Cell morphology analysis was performed by using cell fluorescent images with a diffuse staining. ImageJ 1.53t software package was used. To quantify cell shape, the non-fluorescent background was subtracted from the image, and separate cells were analyzed by choosing a field of region of interest. 50 cells from each cell culture were taken for the analysis. Cell size, aspect ratio, and roundness were analyzed. To compare the values between cell groups, non-parametric ANOVA tests (Kruskal-Wallis test and Dunn’s multiple comparisons test) were performed.

### 4.7. Physical and Nanomechanical Cell Analysis

Cell line analysis was performed via microscope Bruker BioScope Resolve in a semi-contact mode. We used single pre-calibrated silicone-nitride PFQNM-LC-A-Cal probe (Bruker, Billerica, MA, USA) with estimated spring constant 0.084 N/m, tip radius 70 nm. A non-contact thermal noise-based calibration was performed to calculate probe deflection sensitivity and cantilever resonance frequency which were estimated as 17.86 nm/V and 21.74 kHz, respectively [65].

Scanning was done under constant temperature of 37 °C with CO_2_-independent media (DMEM-F12, 10% FBS, 15 mM HEPES). To prevent evaporation, special lids were used. To prevent cell damage and provide good force curves quality during AFM scanning tip, velocity was limited to 66 um/s, peak force tapping frequency was 0.5 kHz, image scan size was 100 um, number of samples per line was 128, and number of lines was 128.

Force curves analysis was carried out by DMT-model (Derjagin, Muller, Toropov model [66]), which is required to analyze sample deformation by amount less than probes radius. Cells’ Young’s modulus and stiffness were calculated as well.

In the IDH1 wild-type culture group, 102 cells were analyzed by AFM. IDH1 R132H cell culture group was analyzed to reach 150 cells investigated by AFM.

For nanomechanical analysis, we used NanoScope analysis software (Bruker, Billerica, MA, USA), which was supplied with the atomic force microscope, and then the data were analyzed via GraphPad Prism 8 (GraphPad Software, San Diego, CA, USA) for group analysis (parametric and non-parametric statistics).

### 4.8. Statistical Analysis

Statistical analysis was performed via GraphPad Prizm 8 (GraphPad Software, San Diego, CA, USA). 

The growth curves were built and analyzed by using the method of least squares regression and doubling times were represented as parameters and 95% confidence intervals.

## Figures and Tables

**Table 1:** Glioma primary cell cultures derived from the patients’ samples.

Cell Culture	Diagnosis	Gender	Tumor Sample IHC Assay Results	IDH1 R132H Mutation Status
BT1	Grade IV glioblastoma	female	GFAP up to 90% cells	wild-type
			Synaptophysin up to 40% cells	
			KI 67 up to 15% cells	
			P53 up to 60% cells	
BT40	Grade IV glioblastoma	female	GFAP up to 80% cells	wild-type
			Synaptophysin up to 40% cells	
			KI 67 up to 10% cells	
			P53 up to 60% cells	
BT32	Grade III astrocytoma	male	GFAP up to 100% cells	mutant-type
			Synaptophysin up to 100% cells	
			Vimentin—negative	
			KI 67 up to 10% cells	
			P53 up to 50% cells	
BT39	Grade III astrocytoma	female	Synaptophysin up to 50% cells	mutant-type
			Vimentin—negative	
			KI 67 up to 10% cells	
			P53 up to 50% cells	

## Data Availability

Not applicable.
